# Oscillations, Intercellular Coupling, and Insulin Secretion in Pancreatic β Cells

**DOI:** 10.1371/journal.pbio.0040049

**Published:** 2006-02-14

**Authors:** Patrick E MacDonald, Patrik Rorsman

## Abstract

Insulin is a potent metabolic regulator that is released by pancreatic beta-cells, which respond to body glucose concentrations. Here the authors explain the physiological basis of insulin release.

It's easy to say we are what we eat, but this simple statement belies the complexity of metabolic signalling that goes into balancing food intake with energy expenditure. One hormone in particular—insulin—is a critically important regulator of whole body energy metabolism. It is secreted from the pancreas when blood glucose levels are high, and it acts to maintain glucose homeostasis by promoting glucose uptake and storage in muscle, fat, and liver. When insulin secretion is absent or reduced, or when peripheral tissues fail to respond to insulin, the result is hyperglycaemia leading ultimately to diabetes. Diabetes affects more than 170 million people worldwide and is associated with several long-term complications including nerve damage, kidney failure, microcirculatory impairment, and a greater risk for heart disease and stroke.

There are two types of secretion: exocrine and endocrine. In endocrine secretion, the secreted molecules end up in the blood and they reach their target cells throughout the body via the circulation. By contrast, exocrine secretion does not involve the circulation and the products are released directly into the outside world. Most of the pancreas serves the exocrine function of secreting digestive enzymes into the gut. Less than 1% of the pancreatic tissue is devoted to an endocrine function. The endocrine tissue of the pancreas is organized as cell clusters, called the islets of Langerhans, which are dispersed throughout the pancreatic exocrine tissue and receive a rich vascular (blood vessel) supply ( Figure 1). A pancreatic islet comprises three main cell types. Pancreatic α cells (15%) occupy the islet periphery and secrete glucagon in response to low blood glucose. Glucagon opposes the actions of insulin, thereby increasing circulating glucose levels. Pancreatic δ cells, the least abundant cell type (5%), are dispersed throughout the islet and secrete somatostatin, which has important paracrine effects that suppress insulin and glucagon secretion. The insulin-secreting β cells are the most abundant cell type (80%) and comprise the islet core.

**Figure 1:**
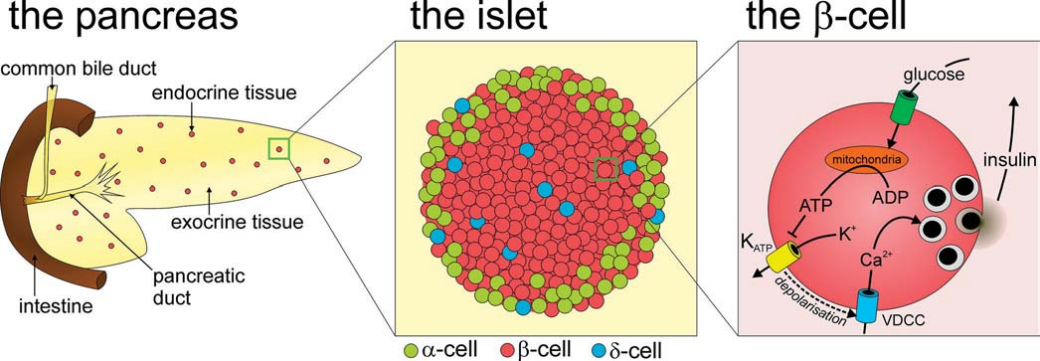
Pancreatic Endocrine Tissue Comprises 1%, or Less, of the Pancreas and Is Organized as Clusters of Cells Dispersed throughout the Exocrine Pancreas These cell clusters, the islets of Langerhans, are heterogeneous and composed of three main cell types that secrete distinct hormones. The majority of islet cells comprise insulin secreting β cells and act as glucose sensors, releasing insulin in response to increased circulating glucose. The mechanism controlling regulated insulin secretion from β cells is shown in the right panel.

During development, the pancreas arises as an off-branching of early gut tissues, and develops as a set of branching tubules which give rise to clusters of endocrine and exocrine cells. Studies have shown that the cytokine TGF-β plays a major role in the development of pancreatic β cells during development of the organ [ 1, 2], and a paper by Smart et al. in this issue of *PLoS Biology* [ 3] demonstrates that TGF-β signalling is also critical in the maintenance of β cell functional identity in the adult. Smart and her colleagues were able to show that loss of TGF-β signalling in these cells causes reversion of these cells to an immature differentiated state and resulted in diabetes. Therefore, TGF-β is important for maintaining the functional characteristics of β cells.

In type 1 diabetes, the less common but more severe form of the disease, pancreatic β cells are destroyed by an autoimmune reaction. Type 2 diabetes accounts for greater than 85% of the cases of diabetes. In this form of the disease, the β cells persist, but for reasons that remain to be established they fail to secrete insulin in sufficient quantities to maintain blood glucose within the normal range. Disrupted insulin secretion is observed prior to onset of type 2 diabetes [ 4], and when combined with the development of insulin resistance in peripheral tissues, results in chronic hyperglycaemia. Further deterioration of β cell function contributes to the progression of type 2 diabetes [ 5]. Type 2 diabetes is believed to result from an unfortunate combination of variants (polymorphisms) in several diabetes susceptibility genes [ 6]. Rarer monogenic forms of the disease result from mutations in genes encoding proteins that are critical to glucose-sensing in the β cell [ 7]. Thus, an appreciation of the mechanisms regulating β cell function and insulin secretion is crucial towards understanding the pathogenesis of type 2 diabetes.

## The Stimulus-Secretion Coupling Mechanism

Glucose-dependent insulin secretion from β cells, by analogy to excitation–contraction coupling in muscle, is referred to as stimulus-secretion coupling. Indeed, like muscle activation, the secretion of insulin is dependent on electrical activity and calcium, Ca ^2+^, entry. β cells have channels in their membranes that allow for the flow of ions (mainly calcium, Ca ^2+^, and potassium, K ^+^) into and out of the cell. Because ions are electrically charged, their flux across the membrane may give rise to sharp changes in voltage (action potentials). Glucose stimulation elicits depolarisation of the cell membrane and electrical activity in β cells [ 8–10]. This serves to open Ca ^2+^ channels in the membrane that respond to changes in voltage—voltage-dependent calcium channels (VDCCs)—and allow Ca ^2+^ entry and action potential firing. Ca ^2+^ acts on the exocytotic machinery to stimulate fusion of insulin-containing vesicles with the plasma membrane for secretion into the bloodstream [ 11]. Removal of extracellular Ca ^2+^ prevents action potential firing [ 12] and insulin secretion [ 13, 14]. Numerous subsequent studies have confirmed the essential roles of glucose-stimulated membrane depolarisation, action potential firing, and entry of Ca ^2+^ in the regulation of insulin secretion.

Metabolism of glucose is essential for insulin secretion, and inhibition of mitochondrial metabolism blocks insulin secretion [ 15]. Mechanisms of β cell glucose metabolism and metabolic signal generation have been recently reviewed [ 16]. The breakdown of glucose results in the generation of ATP, one of the key molecules fueling cellular reactions. An increased ATP:ADP ratio represents the critical link between mitochondrial metabolism and insulin secretion through its ability to close ATP-dependent K ^+^ (K _ATP_) channels and depolarise the cell [ 17] ( Figure 1). K _ATP_ channels are composed of four pore-forming subunits (K _ir_6.2 in β cells) and four accessory sulfonylurea receptor subunits (SUR1 in β cells). The latter are the target of the anti-diabetic sulphonylurea drugs which stimulate insulin secretion by mimicking the effect of glucose to close K _ATP_ channels. Polymorphism in K _ATP_ subunits contribute to diabetes susceptibility by altering the biophysical properties of the channels [ 6].

Under low glucose conditions, K _ATP_ channels are open, allowing the outward flux of K ^+^ and holding the cell membrane potential at about −70 mV. Closure of K _ATP_ channels, by glucose-induced increases in ATP, drives the membrane voltage to more positive potentials, and eventually triggers the firing of action potentials resulting from activation of VDCCs ( Figure 1). The major VDCC subtype expressed in β cells and that regulates insulin secretion is the L-type Ca ^2+^ channel (Ca _v_1.2). The essential role of this channel has been demonstrated both by pharmacological [ 18] and genetic [ 19] inhibition of the channel. Both of these approaches result in a severe reduction in glucose stimulated insulin secretion. Although the L-type Ca ^2+^ channel certainly plays a primary role in the regulation of insulin secretion, it is not the only VDCC expressed in β cells, and recent work suggests an important role for the R-type Ca ^2+^ in insulin secretion during prolonged stimulation [ 20].

## (Un)Coupling Glucose Metabolism and ATP Production in β Cells

Because the membrane voltage is sensitive to changes in ATP levels within the cell, perturbations of the metabolic pathways that generate ATP can have a strong effect on insulin secretion. ATP is generated in mitochondria through the electron transport chain, and is dependent upon the presence of a proton gradient (H ^+^) across the mitochondrial membrane. In β cells, expression of uncoupling protein-2 (UCP2) can disrupt the generation of ATP in mitochondria by permitting protons to leak across the mitochondrial membrane. When UCP2 is overexpressed, the generation of ATP is bypassed [ 21], while loss of UCP2 expression results in increased ATP levels and also enhanced insulin release by islets [ 22]. Accordingly, there may exist a correlation between expression levels of UCP2 and diabetes or obesity.

Although UCP2 clearly plays a role in regulating ATP production, the molecular pathways controlling its expression are not well understood. Bordone et al. (in a paper published in this issue of *PLoS Biology* [ 23]) uncovered one potential regulator of UCP2 expression in their studies of Sirt1 expression and function in murine islets. The authors found that Sirt1, a homologue of Sir2 (which itself is known to play diverse and important roles in regulating metabolism in organisms from yeast to mammals) is expressed in β cells, and that it downregulates UCP2 expression in these cells. This identifies Sirt1 as a positive regulator of insulin secretion from β cells.

## Oscillatory Responses and Cell-to-Cell Coupling in β cells

**Figure 2:**
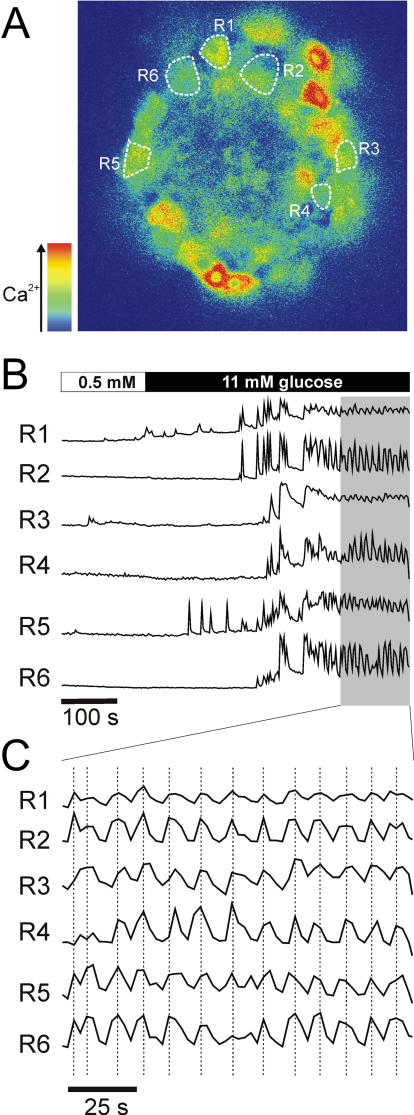
The Responses of β Cells within Intact Islets Are Oscillatory and Synchronised Here, the intracellular Ca ^2+^ responses were measured using ratiometric methods and confocal microscopy. In islet β cells, marked R1-R6 in (A), glucose-stimulation results in increases in intracellular Ca ^2+^ as shown in (B). Oscillations in intracellular Ca ^2+^, with a period of ∼10 s, are observed. Furthermore, as seen in the expanded time scale in (C), these oscillations are synchronized within separate β cells throughout the islet.

Over the physiological range of glucose concentrations, β cell electrical activity consists of oscillations in membrane potential between depolarised plateaux, on which bursts of action potentials are superimposed, separated by repolarized electrically silent intervals. These oscillations in electrical activity are accompanied by changes in the cytoplasmic Ca ^2+^ concentration [ 24], as demonstrated in Figure 2, which in turn give rise to brief pulses (∼10 s) of insulin secretion [ 25–27].

These oscillations reflect a balance between activation of VDCCs (depolarization) and K ^+^ channel activity (repolarization) [ 10]. The depolarizing component predominates at the beginning of the burst, but the resultant influx of Ca ^2+^ during the plateau leads to a progressive Ca ^2+^-induced increase in K ^+^ channel activity. This occurs both via a direct effect on small conductance Ca ^2+^-activated K ^+^ (SK) channels [ 28], and via an indirect effect on K _ATP_ channels by lowering of the cytoplasmic ATP:ADP ratio due to increased Ca ^2+^ ATPase activity [ 29]. The increase in K ^+^ channel activity eventually becomes large enough to repolarize the β cell, ending the burst. In this scenario, the slow pacemaker depolarization between two successive bursts results from the gradual restoration of [Ca ^2+^] _i_ and the ATP:ADP ratio until SK and K _ATP_ channels are again closed and the background depolarizing conductance becomes sufficiently large to trigger a new burst of action potentials.

Glucose produces a concentration-dependent increase in the duration of the bursts at the expense of the silent intervals until eventually, at glucose concentrations beyond 20 mM, uninterrupted action potential firing is observed. This may result from the higher rate of glucose metabolism at high concentrations of the sugar so that Ca ^2+^ influx is unable to lower ATP sufficiently to produce an increase in K ^+^ conductance large enough to trigger membrane repolarization. This model is supported by the ability of tolbutamide, a blocker of the K _ATP_ channel that has been used for more than 50 years to treat diabetes, to suppress β cell membrane potential oscillations that results in continuous firing [ 29, 30]. Thus, the role of K _ATP_ channels in the β cell extends beyond merely serving as the glucose-regulated resting conductance. They also contribute to the progressive stimulation of electrical activity and insulin release by supra-threshold glucose levels.

There is an interesting dependence of oscillatory electrical activity on islet integrity and the 10–15 s period typically observed in intact pancreatic islets is for the most part lost in isolated cells maintained in short-term tissue culture [ 30]. This has been attributed to changes in channel expression [ 30], loss of paracrine signalling [ 31], and requirement of cell coupling [ 32]. Indeed, β cells within the same pancreatic islet are electrically coupled [ 33, 34], such that the [Ca ^2+^ ] _i_ oscillations within different parts of the islet occur in phase ( Figure 2). This synchronization presumably accounts for the observation of pulsatile insulin secretion from individual pancreatic islets [ 26]. Pancreatic β cells contain the gap junction protein connexin-36, ablation of which leads to loss of oscillatory insulin secretion, whereas [Ca ^2+^] _i_ oscillations in the individual cells is maintained [ 35].

Whereas β cells are electrically coupled to each other, electrical coupling [ 36] and synchronization of the [Ca ^2+^ ] _i_ oscillations [ 37] between β cells and non β cells and between non β cells appears much weaker if it exists at all. This it at variance with some of the early data looking at the flow of an injected dye between cells which demonstrated the existence of both homotypic (i.e., β to β cell) and heterotypic (e.g., β to a cell) cell coupling [ 38, 39]. However, more recent observations using noninvasive techniques suggest that dye coupling may be less extensive than previously thought [ 40].

In this issue of *PLoS Biology*, Rocheleau et al. [ 41] have studied the functional significance of electrical coupling between β cells using a novel and ingenious approach. They have used genetically engineered mice in which the K _ATP_ channel is rendered non-functional—by replacement of specific amino acids—in only some of the pancreatic β cells. This mosaic expression of the transgene (Kir6.2[AAA]) results in functional K _ATP_ channel knockout in ∼70% of the β cells. Somewhat surprisingly, intact islets from mice expressing the transgene exhibited an essentially normal glucose-dependent insulin secretion, when tested in vitro. Importantly, this required the integrity of the pancreatic islet since normal glucose regulation was lost upon dispersion of the islet into single cells. Insulin secretion from individual Kir6.2[AAA] islet cells occurred already at 1 mM glucose, which in normal cells is a non-stimulatory concentration. Moreover, insulin release was not further stimulated with increasing glucose concentrations. The observation that application of the gap junction inhibitor 18a-glycyrrhetinic acid to intact islets mimicked the effect of islet dispersion makes it likely that this difference results from electrical coupling that can only operate within the intact islet.

These data are consistent with the view that the islet functions as a syncytium (that is, an organ that in electrical terms behaves like one cell) where K _ATP_ channel activity in the individual cells determines the excitability of the entire organ. This is reminiscent of the channel sharing concept originally proposed by Sherman et al. [ 42] to explain the membrane potential oscillations in islets. Work on isolated cells, even when taken from the same animal, indicate a significant heterogeneity in the time courses and magnitude of their responses to glucose stimulation. It seems possible that this reflects a metabolic heterogeneity and that some cells metabolise glucose better than others. This metabolic heterogeneity will result in variable K _ATP_ channel activity in the individual cells. The report by Rochelau et al. is significant also in this context. They show that all cells within an intact islet respond to glucose in the same way although the K _ATP_ channel activity in the individual cells ranged between zero and 100% of the normal. The only deviation from normality was a slight shift (∼2 mM) towards lower concentration in the glucose dose-response curve. Thus, a lowered K _ATP_ channel activity in the Kir6.2[AAA] expressing cells will increase excitability in their normal neighbours and vice versa.

Can cell coupling be of pathophysiological significance? Given that most of the ATP required for β cell function is of mitochondrial origin, processes that interfere with oxidative phosphorylation are likely to be important in the aetiology of type 2 diabetes. Heteroplasmy of mitochondrial gene mutations leading to lowered ATP production (reviewed by [ 43]) and increased K _ATP_ channel activity in a minority of the β cells within the cell may thus, via cell coupling, compromise electrical activity and secretion in the entire islet, perhaps enough to result in clinical diabetes.

## Abbreviations

UCP2: uncoupling protein-2
VDCC: voltage-dependent calcium channel
